# Dietary practice and nutritional status of low-income earners in a rural adult population in Delta State, Nigeria: a cross-sectional study

**DOI:** 10.11604/pamj.2024.48.138.40722

**Published:** 2024-07-29

**Authors:** Ogbolu Nneka Christabel, Esegbue Peters, Agofure Otovwe, Okonkwo Browne, Aduloju Akinola Richard

**Affiliations:** 1 Department of Public and Community Health, College of Medical and Health Sciences, Novena University, Ogume, Delta State, Nigeria,; 2 Department of Public Health, Faculty of Health Sciences, Achievers University, Owo, Ondo State, Nigeria

**Keywords:** Nutritional knowledge, dietary habits, dietary condition, low-paid workers, diet, body mass index

## Abstract

**Introduction:**

due to the inability of low-income populations to access nutritious foods or basic education, these groups usually consume unhealthy diets, which frequently lead to nutrition issues like obesity, malnutrition, and other health morbidities. The purpose of the study was to evaluate the nutritional knowledge, dietary practices, nutritional status, and factors influencing the dietary habits of low-income persons living in a rural constituency in Southern Nigeria.

**Methods:**

a cross-sectional study was carried out on 419 consenting low-income adults (18 years and older) using a simple random technique, in order to collect data on their socio-demographic traits, nutritional knowledge, dietary practices, and nutritional status. Statistical Package for Social Sciences (SPSS) version 22.0 was used to analyze the data generated.

**Results:**

the respondents´ the average age was 40.9 ± 15.68 years while 224 (53.5%) of those surveyed were females. The proportion of responders with a secondary education was highest 279 (66.6%). The most common occupation among respondents was farming 151 (36.1%) and petty trading 135 (32.2%). Overall, 314 (74.9%) of low-income adults had poor dietary habits, and 245 (60.6%) had poor nutrition knowledge. Occupation and gender were significantly associated with nutritional status P<0.05. The majority of respondents 56.2% (235) were overweight or obese, and multivariate logistic regression analysis shows that respondents with concern about gaining weight were more likely to be overweight or obese (OR=1.065, 95% CI=0.832-1.363).

**Conclusion:**

the findings from the study indicate that inadequate nutritional knowledge and poor dietary habits, reflected in respondents' body weight are likely to increase the risk of non-communicable diseases, necessitating the need for nutritional education among rural populations.

## Introduction

The low-income group according to the World Health Organization [1], are those who earn an average of 446,882 naira or an equivalent of $1045 annually while the Federal Republic of Nigeria (1991) defined it as all wage earners and self-employed persons whose annual income in Nigeria is 20% or less of the highest wage grade level's annual maximum income at any given period [2]. Despite global wealth increase, health disparity persists in low-income countries, worsening obesity and malnutrition. According to World Health Organization [1] low-income families (those with an annual or less earning of $1045) consume unwholesome diets than those who earn $12745 or more annually because they barely can afford enough wholesome foods or lack the privilege of basic education which often leads to nutritional disorders like obesity, malnutrition and other health morbidities [3]. Kennedy *et al*. [4] highlight the lack of fresh fruits and vegetables for a healthy diet in both lower and middle-class families. Low-income communities often sell produce for money, leading to less consumption. Poor nutrition contributes to chronic illnesses like cancer and diabetes, with non-communicable diseases accounting for over 60% of global deaths, with 80% occurring in low-income countries [5,6].

Impoverished communities in countries with low and middle levels of income have been reported to experience a higher burden of chronic non-communicable diseases (NCDs) and nutrition-related NCDs [7]. Nigeria has a stunning rate of 37% nutritional indices which is less than global average making it the world´s second highest. The intake of unhealthy diet and other unhealthy dietary components has caused the rapid emergence of NCDs and the frequency of malnutrition in all its manifestations has been attributed to deficient dietary components, food insecurity, poor socio-economic status, and poor childhood feeding practices among others [8]. In Nigeria, low-income households consume too many calories from staple foods like cassava, rice, yam, and maize and few calories from non-staple foods such as fruits and vegetables, dairy products among others and dependency on these traditional foods has led to nutritional deficiencies as well as NCDs associated with overweight, obesity, diabetes in addition to other cardiovascular diseases [5]. As such, having an understanding of the dietary pattern of low-income households is necessary for the planning and implementation of food and dietary requirement programs [9]. Therefore, the study aimed to determine the dietary practice and nutritional status of low-income earners in a rural adult population in Ndokwa and Ukwuani Local Government Areas of Delta State Nigeria.

## Methods

**Study design and setting:** the study employed an exploratory cross-sectional study design that determined the nutrition-related knowledge, dietary practice, and nutritional status of low-income earners in Ndokwa and Ukwuani Local Government Areas of Delta State in Nigeria. Ndokwa/Ukwuani consists of 3 Local Government Areas namely Ndokwa East, Ndokwa West, and Ukwuani. Ndokwa lies between latitudes 50 48´´ N and 50 60´´N and longitudes 60 08´´ E and 60 32´´E of Delta State [10].

**Study population:** the study population consists of low-income earners in the three local governments that make up Ndokwa/Ukwuani Local Government Area of Delta State. According to the study, low-income earners are those self-employed population male or females which include subsistence farmers, petty traders, vulcanizers, labourers, all those with small scale-sized businesses and without a defined means of livelihood whose annual income is 20% or less of the highest wage grade level's annual maximum income at any given period [2]. The study's inclusion requirements include living in the study area, being self-employed, engaging in subsistence farming, small-scale trading, vulcanization, labour, and any other small-scale business without a defined source of income whose annual income is 20% or less of the highest wage grade level's annual maximum income at any given time. Additionally, participants cannot participate in the study if they have not given their consent. All those who did not meet the inclusion criteria and did not provide consent to participate in the study were excluded from the study.

**Sample size and sample technique:** the Yaro Yamane formula was applied to calculate the sample size based on a 95% confidence level, 5% error horizon, and given the total population of age 20 and above in Ndokwa/Ukwuani Local Government Area to be 189,217 in line with the 2006 population census [11-13].


$$n=\frac{N}{1+N{\left(e\right)}^{2}}$$

Where n= sample size; N= population size; e= level of precision or confidence interval ±5% = 0.05; to calculate for Ndokwa/Ukwuani federal constituency with population size 189,217.


$$\begin{array}{lll} n & = & \frac{189217}{1-189217{\left(0.05\right)}^{2}}\\ n & = & \frac{189217}{469.0425}=399.183 \end{array}$$

To attribute for the 10% non-response and attrition rate, the size of the sample was adjusted to 444 only 419 responded to the questionnaires giving a response rate of 94.36%. To achieve the number of questionnaires that were distributed across the LGAs, a simple random technique of balloting was employed to choose a community each in the three LGAs. A multi-stage sampling procedure was applied to choose respondents.

**Stage one: selection of local government area:** Delta State was clustered into 3 senatorial districts which are Delta North, South, and Central. Delta North was randomly picked by balloting and was further stratified into 9 LGA. Ndokwa East, West, and Ukwuani Local Government Area were randomly selected.

**Stage two: selection of communities:** Ukwuani, Ndokwa East, and West LGAs consist of 9, 10, and 7 communities respectively. Using a simple random approach by balloting method, Amai, Aboh, and Abbi were selected.

**Stage three: selection of quarters:** Amai, Aboh, and Abbi consist of 5, 5, and 3 quarters respectively. Three-quarters were selected in each community. These quarters were Umuekum, Ishikaguma, and Umuosele of Amai in Ukwuani LGA, Ekwelle, Umeai and Elovei of Abbi in Ndokwa East, and Umu-Ujugbeli, Umu-Ossai, and Umu-Obi of Ndokwa West.

**Stage four: selection of houses:** all the houses in the three-quarters in each selected community were used for the study.

**Stage five: selection of households:** only one household (family) in each of the houses in each quarter that were selected across the 3 LGAs was used for the study. If there are two or more households in a house, then the random selection by balloting technique process was adopted to pick a household.

**Stage six: selection of respondents:** only respondents that met the inclusion criteria (all petty traders, subsistence farmers, and low-scale artisans who were above 18 years) in a household were selected and if there were more than one eligible respondent, only one was chosen using the balloting process.


$$\text{Distribution of questionaries across the }3\text{ LGAs}=\frac{\text{Total pop in LGA from age}\left(20-100\right)\times \text{sample size}}{\text{Total pop in }3\text{ LGA}}$$

Ukwani = 60,440 x 444 ÷ 189,217 = 142, since 3 quarters were recruited for the research, 142 ÷ 3= 47. Therefore, 47 questionnaires were given out in the three quarters selected in Amai, Ukwuani LGA. Ndokwa East = 52,524 × 444 ÷ 189,217 = 123, 123 ÷ 3 = 41. Therefore, 42 questionnaires were given out in the three quarters selected in Aboh of Ndokwa-East LGA. Ndokwa-West = 76,253 × 444 ÷ 189,217 = 178, 178 ÷ 3 = 59. Thirty-three questionnaires were given out in the three quarters selected in Abbi of Ndokwa-West LGA.

**Method and instrument of data collection:** data collection was performed utilizing a detailed semi-structured questionnaire from August 2021 to March 2022. The questionnaire was divided into 4 sections: a socio-demographic characteristic which includes gender, marital status, age occupation, degree of education, and a number of persons in the household, an adapted food and nutrition-related knowledge and dietary practice questions developed by the Food and Agriculture Organization of the United Nation (FAO-UN) [14] and from a previous study [15]. An electronic weighing scale set to the nearest 0.1 kilogram (HANA, Big Boss, China) was used to take their weight, and heights were measured to the closest meter using a tape to be able to calculate BMI according to World Health Organization standard [16] and a section on the variables influencing their food decisions. Information on the frequency of meals-such as the number of breakfasts, lunches, and dinners as well as the practice of skipping meals were obtained. Other information gathered includes information on food preservation techniques, intake of fruits, vegetables, sugar-sweetened beverages, cereals, seafood, beans, and alcohol.

**Definitions:** knowledge of nutrition: this speaks of people's knowledge and understanding of many aspects of diet and the effects they have. Dietary practices: this comprises patterns of food intake (frequency and types consumed), meal frequency, food sources (homemade versus bought) and status of food security. Nutritional status: the state of a person's health as it relates to the consumption and use of nutrients is referred to as their nutritional status. This may be assessed using the Body Mass Index (BMI) metric, which calculates body fat based on height and weight. Low-income earners: the low-income earners in Ndokwa/Ukwuani Local Government Areas that were utilized for this study included all subsistence farmers, petty traders, vulcanizers, labourers, all those with small-scale sized businesses, and without a defined means of livelihood.

**Statistical methods:** the retrieved data was analysed with Statistical Package for Social Sciences version 22.0 (IBM Corp, Armonk, NY, USA). Variables were described in descriptive statistics (frequencies and percentages) and also presented in tables. Pearson's Chi-Square test was utilized to check for statistical variations between the dependent and independent variables. The dependent variable was the nutritional status of low-income earners while the independent variables were their age, gender, occupation, dietary practices, and knowledge. The level of significance for the test of significance was established at P< 0.05, with a 95% confidence range. Logistic regression was used to predict the factors that could influence their dietary practice.

In the scale of measurement, 6 questions were scored on a 12-point weighted scale. Each accurate response received two marks, while non-correct responses received zero. According to scoring and categorizing, respondents with 7 or higher ratings of knowledge were deemed to have strong knowledge, while those with 7 or lower ratings of knowledge were deemed to have poor knowledge. Dietary practice was scored on a 14-point scale and respondents with scores of 7 or higher were thought to have good dietary habits, while those with scores of 7 or lower were seen to have poor dietary habits.

**Ethical approval:** the Department of Public and Community Health in Novena University, Delta State, first gave permission for the research to be carried out as well as ethical clearance from the Research Ethics and Grant Committee, Delta State University with reference number RBC/FNMC/DELSU/24/330. Following a satisfactory explanation regarding the study, participants gave consent to participate by appending their signature on the questionnaire administered. Additional authorization was secured from the respective community heads who provided letters of approval to that effect.

## Results

**Socio-demographic characteristics of respondents:** majority 247 (58.9%) of those surveyed were between ages 20-40, the least 58 (13.8%) were 61 years above and the majority were females 224 (53.5%). Nearly all the respondents 279 (66.6%) attained a secondary education level while the least number of respondents 11 (2.6%) lacked academic training. Among the respondents, 151 (36.0%) were subsistence farmers which means farming was their major source of income and 135 (32.2%) were petty traders (Table 1).

**Table 1:** socio-demographic characteristics of the respondents (n=419)

Variables	Frequency	Percentage
**Age (40.9 ± 15.68 years)**		
20-40	247	58.9
41-60	114	27.2
61 above	58	13.8
**Gender**		
Female	195	46.5
Male	224	53.5
**Level of education**		
Primary	57	13.6
Secondary	279	66.6
Tertiary	72	17.2
No formal education	11	2.6
**Occupation**		
Subsistence farming	151	36.1
Petty traders	135	32.2
Artisans	133	31.7

**Nutritional knowledge and dietary practice of respondents:** the vast majority of participants 338 (80.7%), were unaware of the various food classifications, whereas only 81 (19.3%) were. Two hundred and ninety-six people, or 70.6%, were unaware what a balanced diet was, while only 123 people, or 29.4%, said they understood what it meant. One hundred and eighty-nine people (45.1%) and 230 people (54.9%) respectively knew what a three-square meal was. Furthermore, 393 respondents (93.8%) out of the total respondents knew that diet plays a significant role in disease prevention and management, with 26 respondents (6.2%) making up the smallest percentage. Additionally, a higher proportion of respondents-341 (81.4%)-knew that adults should consume more fruits and vegetables in addition to less red meat, carbohydrates, and fats. While 78 (or 18.6%) are ignorant of this information. Two hundred and fifty-two (252) respondents, or 60.9%, said they knew how to preserve their food the best way, while 138 respondents, or 32.9%, said they didn't. Following additional categorization, it was found that among respondents; nutritional awareness was 60.6% poor and 39.4% good (Table 2).

**Table 2:** nutritional knowledge of the respondents

Variable	Frequency	Percentage
**Do you know the different class of food?**		
Yes	338	80.7
No	81	19.3
**Do you know what a balanced diet is?**		
Yes	123	29.4
No	296	70.6
**Do you know what a 3-square meal is?**		
Yes	189	45.2
No	230	54.9
**Do you know that food is important for the prevention of diseases?**		
Yes	393	93.8
No	20	6.2
**Do you know that adults should eat more of fruits, vegetables and less of fats and red meats?**		
Yes	341	81.4
No	78	18.6
**Do you know how to preserve food?**		
Yes	252	60.1
No	138	32.9
**Level of dietary knowledge**		
Good	165	39.4
Poor	245	60.6

Among the respondents, 373 (89.0%) expressed a preference for eating meals made at home, while 243 (58.0%) indicated that all meals are significant to them. The most part of respondents 208 (49.6%) eat twice daily, while 179 (42.7%) claimed they never eat three square meals. While 173 (41.3% of respondents) stated they never ate breakfast, 174 (41.5%) said they occasionally did so. Most of the survey participants, 179 (42.7%), claimed they never consume fruits and vegetables. When asked how often they skip meals, 274 (65.4%) said frequently, compared to 226 (53.9%) who had never preserved their food. Furthermore, 292 (69.7%) reported that they consume processed cereal frequently, whereas 210 (50.1%) confirmed that they frequently consume sugar-sweetened drinks. A large majority of individuals (75.7%) from the survey also reported eating fish infrequently, while the majority of participants (77.3%) reported eating meat frequently. Furthermore, among the respondents, 265 (63.2%) frequently eat beans whereas 173 (41.3%) frequently drink alcohol. In general, dietary practice shows that it was 25.1% good and 74.9% poor among the respondents (Table 3).

**Table 3:** dietary practice of the respondents

Variable	Frequency	Percentage
**Do you enjoy eating homemade meals?**		
Yes	373	89.0
No	46	11.0
**What is the most important meal for you in the day?**		
Breakfast	81	19.3
Lunch	58	13.8
Dinner	27	8.8
All of them	243	58.0
**How many times do you eat daily?**		
1	1	0.2
2	208	49.6
3	131	31.3
4	65	15.5
More than 4 times	14	3.3
**How often do you observe 3 square meals?**		
Very often	89	21.2
Often	5	1.2
Sometimes	146	34.8
Never	179	42.7
**How often do you take breakfast?**		
Very often	70	16.7
Often	2	0.5
Sometimes	174	41.5
Never	173	41.3
**How often do you eat your fruits and vegetables?**		
Very often	67	16.0
Often	5	1.2
Sometimes	168	40.1
Never	179	42.7
**How do you preserve your food?**		
Drying	72	17.2
Refrigerator	42	10.0
Salting	21	5.0
Smoking	54	12.9
Warming/re-heating	3	0.7
None	226	53.9
**How often do you skip meals?**		
Very often	36	8.6
Often	274	65.4
Sometimes	79	18.9
Never	30	7.2
**How often do you consume sugar sweetened drinks?**		
Very often	210	50.1
Often	55	13.1
Sometimes	115	27.4
Never	39	9.3
**How often do you consume processed cereals?**		
Very often	292	69.7
Often	112	26.7
Sometimes	12	2.9
Never	3	0.7
**How often do you consume fish?**		
Very often	97	23.2
Often	4	1.0
Sometimes	317	75.7
Never	1	0.2
**How often do you consume meat?**		
Very often	324	77.3
Often	42	10.0
Sometimes	38	9.1
Never	15	3.5
**How often do you consume alcohol?**		
Very often	149	35.6
Often	173	41.3
Sometimes	60	14.3
Never	37	8.8
**How often do you consume beans?**		
Very often	102	24.3
Often	265	63.2
Sometimes	43	10.3
Never	9	2.1
**Level of dietary practice**		
Good	105	25.1
Poor	314	74.9

**Factors influencing respondent´s dietary practice:** there was no correlation between knowledge and nutritional status, age, and nutritional status at P<0.05 (P=0.951, 0.135), a significant correlation exists between occupation and nutritional status and between gender and nutritional status (P=0.014, P=0.000) respectively. Further results showed factors that affected their dietary practice and multivariate analysis reveals that concern about weight increase (odd ratio=1.065, 95% confidence interval=0.832-1.363) predicted this behaviour (Table 4).

**Table 4:** factors influencing dietary practice of respondents

Variable	Wald	Df	Sig.	Exp(B)	95% C.I for Exp(B) lower	Upper
**Factors influencing dietary practice**						
Income	0.505	1	0.477	0.959	0.854	1.077
Time	2.190	1	0.139	0.826	0.642	1.064
Inadequate information	0..817	1	0.366	0.893	0.700	1.141
Quality of food	11.195	1	0.001	0.647	0.501	0.835
Weight gain	0.252	1	0.616	1.065	0.832	1.363
Culture	14.654	1	0.000	0.663	0.538	0.818
Appetite	0.004	1	0.948	0.990	0.734	1.336

Sig: significance; OR: odd ratio; Df: degree of freedom; Exp (B): exponential value of coefficient B; 95% C.I: 95% confidence interval

**Nutritional status of respondents:** nutritional status reveals that 36% (151) of the participants were overweight, 20.8% (87) were obese, 1.9% (8) were underweight and 41.3% (173) had healthy weight.

## Discussion

The main objective of the study was to determine the dietary practice and nutritional status of low-income earners in a rural adult population in Ndokwa and Ukwuani Local Government Areas of Delta State, Nigeria. The findings of the study showed that the study participants demonstrated poor nutritional knowledge, and exhibited poor dietary habits; more than half of the respondents were either overweight or obese, and nutritional status varied across socio-demographic characteristics.

Unhealthy eating practices and poor diet choices have always been considered an underlying reason for poor health status, especially among low-income populations. According to Azizana *et al*. [17] unhealthy eating practices and poor diet choices among low-income populations have been observed as a prime reason for poor health status.

The research reveals that the majority of respondents are 20-40 years old, with a mean age of 40.9 ± 15.68 years. Females (53.5%) are more likely to live in low-income households than males (46.5%) [18]. Most respondents attended secondary school (66.6%), and the most popular occupations were farming (36.0%) and petty trading (32.2%). This finding is similar to the finding by Afolabi *et al*. [19] that the most prominent occupations of low-income earners are farming and trading.

Furthermore, more than half of the respondents had poor nutritional knowledge, which could lead to poor dietary habits and health issues. Corroborating with this observation is a study by Sun Y *et al*. [20] that revealed that a low level of nutrition knowledge increases the potential for chronic diseases and other health problems however this is contrary to previous studies that found good knowledge among chronic disease patients and among low-income residents, women of childbearing age, and older persons [7,21-23]. Differences in socioeconomic characteristics may contribute to these disparities.

It was shown that individuals who frequently drank soft drinks had poor eating habits, which contributed to weight gain and other health problems. A report by Vartanian *et al*. [24] revealed an obvious connection exists between soft drinks and increased body weight. A high percentage (56.1%) seldom eats fruits and vegetables, this is similar to [17,25] that participants in lower-income households did not consume enough fruits and vegetables necessary for chronic disease prevention. This also corroborates with Heshmat *et al*. and French *et al*. [26,27] that families with high socioeconomic status consume more fruits and vegetables and purchase healthier foods than low-income households. In addition, Leone *et al*. [28] and Huang *et al*. [29], attributed the poor consumption of fresh produce for those on a limited budget to behavioral and psychosocial factors. Overall, respondents practiced unhealthy eating habits, which was similar to the findings of a previous study by Azizana *et al*. [17]. Chronic disorders including obesity, diabetes, cardiovascular disease, hypertension, and cancer are associated with poor dietary patterns, which include processed foods, sugar-filled beverages, and saturated fats. They also cause health disparities, higher healthcare expenses, and dietary deficits.

Factors such as culture, religion, taste, time, price, age, income level, weight concerns, income, and knowledge about dietary habits have been shown to influence low-income consumers' dietary choices [22,30-34]. Income does not deter dietary habits although studies have put forward that higher income is connected with improved diet quality, and diet practices, and vice versa [17,27,35-38]. Howbeit a notable factor revealed to be influencing respondents´ dietary habits was concerned weight gain explaining why over 40% of respondents are overweight or obese, which could lead to non-communicable diseases, increased healthcare costs, loss of productivity, and mortality [39,40].

No correlation was found between respondents' knowledge of dietary habits and their nutritional status (0.951). This finding was consistent with previous studies on mixed economic statuses and middle-income populations, which also found no significant association between dietary knowledge and body mass index [41,42]. This implies that improving nutritional status is not always the result of learning about good eating practices. Occupation and nutritional status among respondents showed a significant correlation (0.000), indicating that jobs had an impact on body mass index. Mechanized farming increases obesity risks due to less physical activity compared to traditional farming [43]. Although farmers are physically active, their BMIs are abnormal, suggesting other factors like diet and lifestyle behaviors contribute to their increased BMI [44]. The extent of occupational physical activity also influences overall energy use [43-46]. The creation of interventions, such as workplace wellness programs or nutritional education, for certain occupational groups, might be guided by the relationship between occupation and nutritional status.

A significant association between gender and BMI, with males having normal weight and females being more obese was found. Both genders were underweight, corroborating previous studies showing females in low-income sub-Saharan countries are more obese than males and this disparity is attributed to biological factors such as menopause, cultural values, occupation, and level of physical activity [47]. This is also congruent with Tantut *et al*. and Muhammad *et al*. [44,48]. However, Chinedu *et al*. [49] conducted a survey on young adults and found that females were more overweight, underweight, and obese than males. This highlights the burden of malnutrition and over-nutrition among both genders. The development of gender-specific treatments that meet the particular demands and difficulties faced by men and women can be influenced by the recognition of gender differences in nutritional status. The study found no significant relationship between respondents' age and their BMIs at 0.135. This contradicts a previous study suggesting that aging can lead to poor nutritional status due to impeded food acquisition, digestion, and metabolism [50]. Lastly, the study accounts that 1.9%, 41.3%, 36%, and 20.8% of participants were underweight, healthy, overweight, and obese respectively indicating that those in good physical shape are less susceptible to weight-related health issues, and low-income populations are more susceptible to overweight and obesity [48,51].

By offering a snapshot of food habits and nutritional health at one particular moment in time, the cross-sectional design makes it possible to evaluate correlations between variables and pinpoint possible intervention areas.

Although data was gathered based on respondents' responses, a limitation of this research could be prone to recall bias.

## Conclusion

Overweight and obesity is quite prevalent in the communities due to poor socio-economic status. The relatively low number of the participants fell within the recommended healthy weight category however overweight and obesity seemed to be very high. Increased healthcare expenses and a larger illness burden may result from the high incidence of these disorders in low-income populations. One significant factor found to influence their dietary habits was concerns for weight gain and this accounts for why more than 40% of them were overweight and obese which increases cardiovascular disease risk. Healthcare professionals and policymakers should promote nutrition education on the consumption of indigenous fruits and vegetables as well as weight management to prevent malnutrition and chronic diseases.

### What is known about this topic

- *Improper diets and dietary components are linked to the rapid growth of non-communicable diseases, often leading to malnutrition in all their manifestations;*
- *Nigerian low-income households are excessively consuming basic and traditional foods, potentially leading to nutritional deficiencies and non-communicable diseases;*
- *Socioeconomic factors like job, education, and housing stability significantly impact low-income individuals' nutritional status and eating habits, potentially hindering their ability to improve their lives.*

### What this study adds

- *The study documents overweight and obesity among low-income populations making them at risk of cardiovascular diseases;*
- *Concern for weight gain was an identified factor that influenced their dietary habits;*
- *Gender plays a significant role in the weight of an individual as women are more likely to be obese and overweight than males.*
